# Introducing the New Surgical Robot HUGO™ RAS: System Description and Docking Settings for Gynecological Surgery

**DOI:** 10.3389/fonc.2022.898060

**Published:** 2022-06-09

**Authors:** Salvatore Gueli Alletti, Vito Chiantera, Giovanni Arcuri, Alessandro Gioè, Riccardo Oliva, Giorgia Monterossi, Francesco Fanfani, Anna Fagotti, Giovanni Scambia

**Affiliations:** ^1^ Unità Operativa Complessa (UOC) Ginecologia Oncologica, Dipartimento per la salute della Donna e del Bambino e della Salute Pubblica, Fondazione Policlinico Universitario A. Gemelli IRCCS, Rome, Italy; ^2^ Obstetrics and Gynecology, Università Cattolica del Sacro Cuore, Rome, Italy; ^3^ Unità Operativa Complessa (UOC) Ginecologica e Ostetricia, Dipartimento Materno-Infantile, Ospedale Buccheri La Ferla Fatebenefratelli, Palermo, Italy; ^4^ Department of Gynecologic Oncology, Aziende di Rilievo Nazionale di Alta Specializzazione Civico Di Cristina Benfratelli, Palermo, Italy; ^5^ Department of Gynecologic Oncology, Università di Palermo, Palermo, Italy; ^6^ Unità Operativa Complessa Tecnologie Sanitarie, Fondazione Policlinico Universitario A. Gemelli IRCCS, Roma, Italy

**Keywords:** robotic-assisted surgery (RAS), gynecological surgery, HUGO™ RAS, radical hysterectomy, docking, robotics

## Abstract

This study provides a detailed description of the new HUGO™ RAS System and suggests docking settings for gynecological surgery. The system is composed of an “open” surgical console with an HD–3D passive display, a system tower, and four arm carts. Each arm has an extremely wide range of adaptability resulting from the numerous joints. The human cadaver labs were performed at the ORSI Academy between August and December 2021. All procedures were performed by two surgical teams, each composed of a high-volume surgeon experienced in robotic surgery, gynecologic oncology, and pelvic sidewall surgery, and one bedside assistant. Three main gynecological surgical scenarios were identified: standard pelvic surgery, pelvic sidewall surgery, and para-aortic/upper abdominal surgery. Concerning the port placement, the chosen options were called “straight” and “bridge”; instead, the so-called “compact” and “butterfly” configurations were identified for the arm cart positioning. Four cadavers were used to perform total hysterectomy, radical hysterectomy, pelvic exenteration, pelvic and para-aortic lymphadenectomy, and omentectomy. We performed several tests, identifying the best system configurations to draw the proper efficiency from the flexibility of the system in all gynecological surgical scenarios. The straight port placement seems to be adequate for standard pelvic surgery. The bridge trocar position is best to reach the deeper and lateral anatomical regions of the female pelvis. The compact and butterfly arm cart allocations are adequate for both straight and bridge port placement. When deep pelvic surgery was performed, the bedside assistant became more proficient by working with a standard laparoscopic instrument from an ancillary port placed in the left iliac fossa. The arm carts needed to be moved in an open manner, like for the proposed butterfly configuration. On the contrary, the compact disposition left enough space to assist from Palmer’s point port. Several basic and advanced gynecological surgical procedures were performed and completed successfully without encountering any technical or surgical issue, the results obtained were judged sufficient to proceed with the clinical experience in daily practice. The HUGO™ RAS system is flexible and highly performative in various surgical scenarios.

## 1 Introduction

The use of laparoscopic and robotic procedures has increased in gynecological surgery in the last 20 years (1). In particular, robotic-assisted surgery has had a rapid technological evolution constantly supported by ever more robust evidence of the main advantages of this approach over laparotomy (2, 3).

The Da Vinci^®^ (Intuitive Surgical) represented the leading actor in defining the “rules” of robotic surgery: a laparoscopic-like tower allocating the energy and light sources, the insufflator, and the “electronic brain” of the system, connecting the driving console with the four robotic arms coming from a unique monolithic structure named boom. However, technology runs fast, and since 2013, new concepts in robotic systems have been introduced to the scientific community.

The Senhance ^®^ surgical system (first called Telelap ALF-X) was launched in the European market in 2013, introducing a new concept of robotic systems. Indeed, for the first time, robotic arms were designed to be independent wheeled units with the possibility of being moved into the operative room, around the operative table, and used in a variable number of ways, from 1 to 3, according to the surgical program. This configuration was devised to bring more “flexibility” to a robotic surgical system. Differently from the past, the surgeon could allocate robotic arms around the patient, customizing the docking on the basis of both abdominal anatomy and surgical procedure. This characteristic made this robotic approach feasible and reproducible in pioneering published papers (4–8). On the other hand, from clinical use, it has emerged that four independent and wheeled robotic arms require space in the operative room (OR) and significant restoration of spaces usually dedicated to the surgical team, including the anesthesiologist and circulating nurse (9).

More recently, a novel robotic system was launched by CMR Surgical: the Versius^®^ surgical system. The company invested in the already mentioned “flexibility,” designing a modular system characterized by bedside units, called BSUs, small enough to be allocated in a standard operative room and easily removed at the end of surgery (10–12). From preclinical and pilot-published studies, it represents both an evolution and a revolution. Evolution because each robotic component has been significantly reduced in size, becoming a discreet presence in the OR. Revolution because the size and the maneuverability of each robotic element make this modular system easily movable from OR to OR, making it an “instrument” that can be chosen to be used accordingly to the needs of patients. That is a nice step in “flexibility.”

In this panorama, the last robotic system introduced by Medtronic is named the HUGO™ RAS System. It is composed of an “open” surgical console with an HD–3D passive display, a system tower, and four arm carts. Each robotic arm is independent and extendible thanks to 6 different joints. It can also be raised or lowered on the cart column for vertical positioning. The robotic arms are designed to be hooked up to the trocar, and the instruments installed are driven by a dedicated motor called the instrument drive unit. Even in this case, the patient can benefit from the movable arm with an extremely wide range of adaptability resulting from numerous joints.

However, every coin has two sides: independent robotic arms and flexibility around the patients, which also mean a rise in variables. Since each robotic arm is a set of parts of fixed length and connected by articulations with limited movements, by considering that the wheel and tip of the instrument represent the two extremes of a robotic arm, it becomes easy to understand the importance, and the complexity, of docking multiple robotic arms surrounding independently the patient instead of coming from a boom. It is said that preclinical experiences are essential to give newly minted user guidance in understanding, interpreting, and properly using the variability of positioning and docking in the interest of a successful surgery.

This study provides an exhaustive description of the new HUGO™ RAS System and suggests docking settings for gynecological surgery.

## 2 Material and Method

The human cadaver labs were performed at the ORSI Academy in Gent, Belgium, between August and December 2021. All human cadavers were fresh-frozen full-body, donated with consent. The studies were conducted in compliance with the ethical regulations in force at the Orsi Academy.

All procedures were performed by two surgical teams, each composed of a high-volume surgeon experienced in robotic surgery, gynecologic oncology, and pelvic sidewall surgery, and one bedside assistant. All surgical team members had completed the official technical training on HUGO™ RAS technology delivered by the company at the time of the experimental session. The robot was installed in a dedicated area, similar to a classical OR. The four-arm carts were allocated around the operative table, the tables of instruments on one side, and the console unit on the other corner.

### 2.1 Robotic System Description

#### 2.1.1 Console Unit

**Figure 1:**
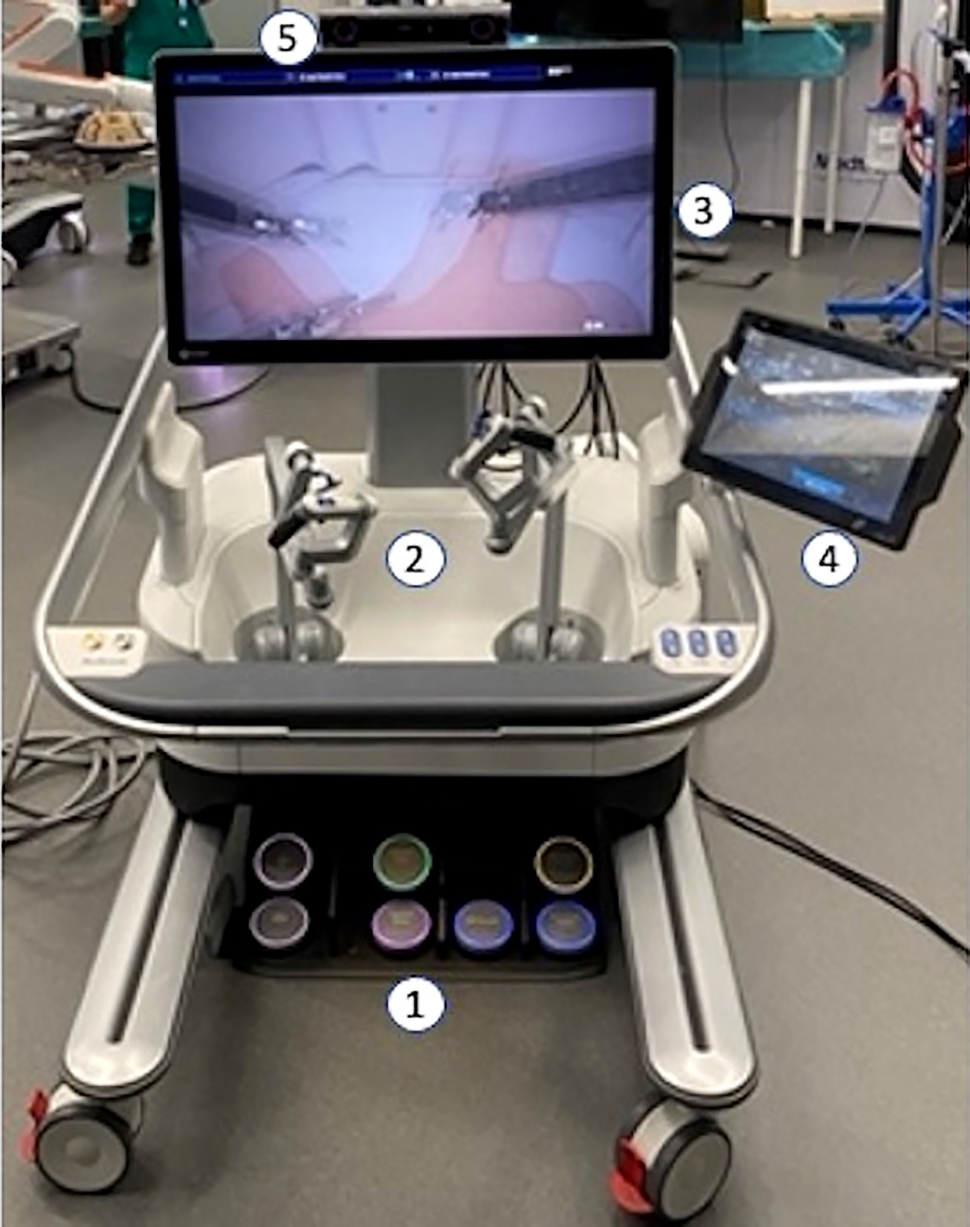
1—Pedal unit; 2—Pistol Grip Manipulators; 3—3D–HD monitor; 4—Surgeon Interactive Display; 5—Head Tracking System.

The surgical console is the driving unit of the entire system (**Figure 1**). Once the surgeon takes place in the chair, the optimal ergonomic position can be achieved by adjusting the position of the viewer, the armrest, and the pedal unit. The pedals control the following functions: the robotic arms and the energy supply (monopolar, bipolar, and LigaSure™); the master clutch allows the repositioning of the manipulators; a switch pedal is located laterally to shift between the 3rd and 4th arm; and finally, the endoscope control pedal allows for repositioning of the endoscope.

To drive the robot, the surgeon handles the “pistol grip” manipulators where infrared contact sensors enable the robotic movement, revealing the hand of the surgeon. On the lower part of each manipulator, a “clutch” button can be pushed to reposition the manipulator itself; on the top, are installed forward and backward buttons to use the robotic endo-stapler. The operating surgeon has two monitors available: a passive 3D–HD screen to see the surgical screen using 3D glasses, and, on the right side, a touchscreen surgeon interactive display to assign the hooked instruments to the right/left hand, set the motion scaling, and select visual filters and camera rotation.

Over the 3D–HD monitor is installed the head tracking system: a vison device that reveals the alignment between reflective markers on the 3D glasses of the surgeon and the surgical monitor, enabling the robotic control. Once this alignment is lost because the surgeon is looking at a point different from the surgical field, the robotic control is disabled.

#### 2.1.2 System Tower

**Figure 2:**
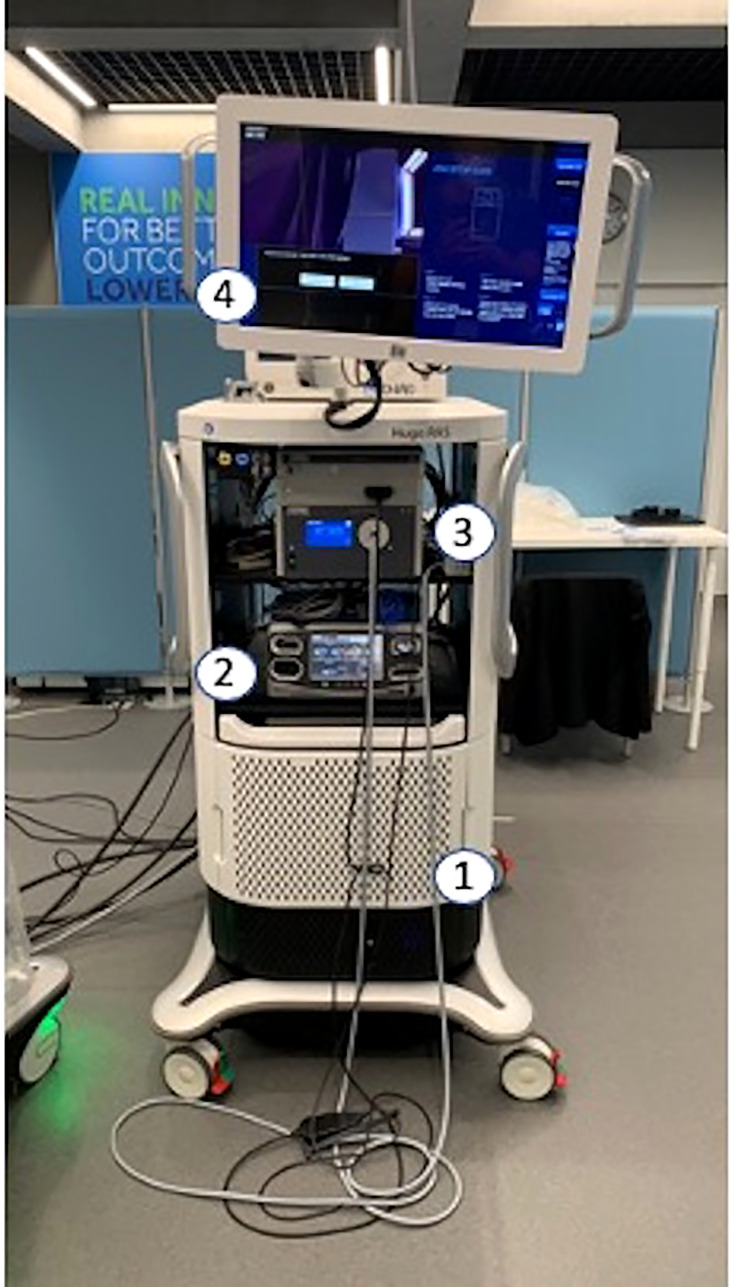
1—Computers and power management; 2—Electrosurgical generator; 3—Vision System; 4—2D Touchscreen Monitor.

The system tower houses computers, the power management system, and a backup battery in the bottom part (**Figure 2**). In the middle are allocated the electrosurgical generator (Covidien AG) and the 3D–HD vision system (Karl Storz). The 2D–HD touchscreen is the interactive display on the top of the operating room team. The system tower, bridging between the console and the arm through single anti-crushing cables, allows the surgeon to control the movements of up to four arms. It may also be used without the surgeon console to power up to four arms for standalone manual control at the bedside, or by itself for standard laparoscopic visualization and electrosurgery.

#### 2.1.3 Robotic Arm

**Figure 3:**
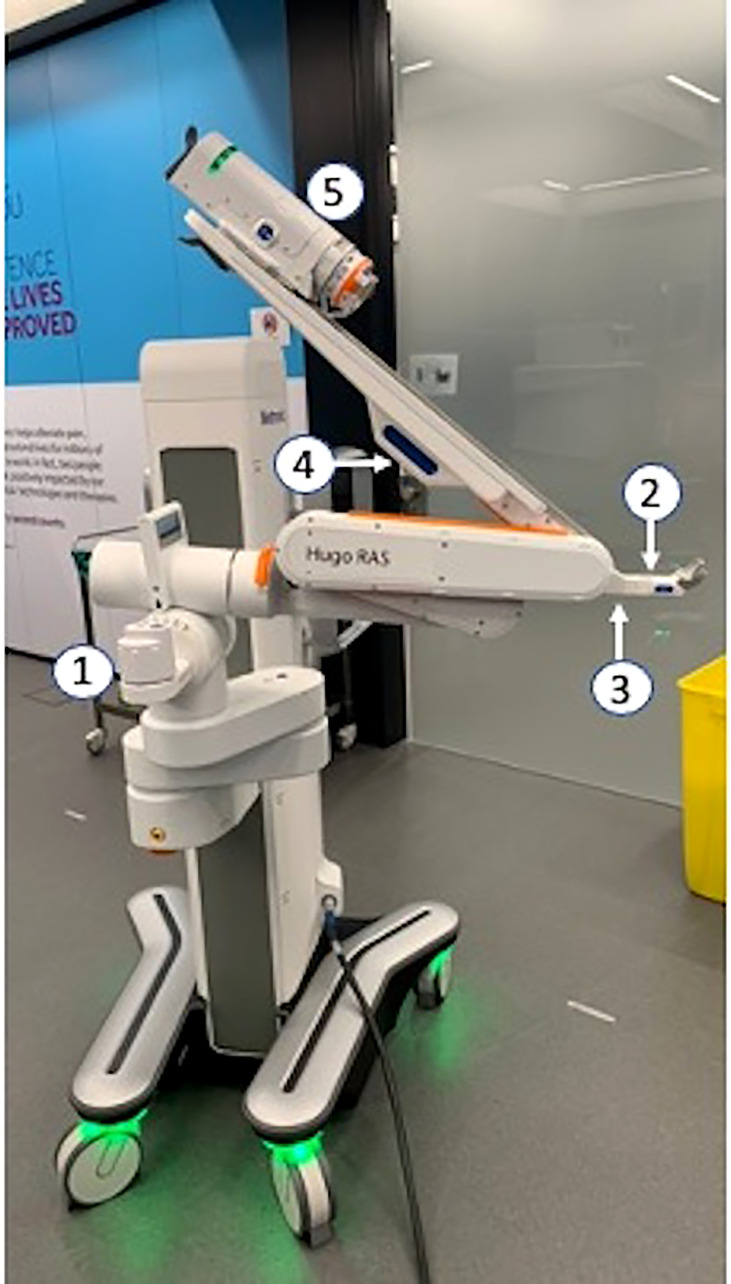
1—Laser Alignment Unit; 2—Position Button; 3—Tilt Button; 4—Elbow Button; 5—Fulcrum Handle; 6—Instrument Drive Unit.

It is the active component that physically replaces the hands of the surgeon in the operating field. In the modern panorama of modular robotic systems, it represents a significant evolution due to the wide range of positioning provided by six different joints (**Figure 3**). Once approaching the operative table, each arm can be raised or lowered on the cart column for vertical positioning. Prior to approaching the patient, the laser alignment unit must be set in parallel with the operative table and oriented with the body (head and legs) of the patient to create a frame of reference in the space for the HUGO™ RAS system. After being done with this, each robotic arm will measure, in a digital space, the angle of approach to the patient. By pushing the “position button,” the “tilt button,” the “elbow button,” and the “fulcrum handle.” the arm can be unfolded, extended, rotated, and hooked to the robotic trocar. Thus, the robotic instrument can be installed on its “drive unite” and inserted into the trocar.

### 2.2 Docking and Surgical Procedures

Based on each basic capability in extension, elevation, and rotation of the robotic arm, the company highlights a standard setup configuration to accomplish a good installation for gynecological procedures. Generally, the trans-umbilical trocar, where the camera is inserted, represents the main landmark for the whole port placement.

However, considering that both the variability of the abdominal conformation and the port-placement represent the entry point of the robot into the abdominal cavity, the surgical plan is the crucial element. Besides the technical and anatomical aspects, the docking needs to be personalized in accordance with the surgical procedure that will be performed.

We identified three main gynecological surgical scenarios to outline these principles: standard pelvic surgery, pelvic sidewall surgery, and para-aortic/upper abdominal surgery.

#### 2.2.1 Standard Pelvic Surgery

**Figure 4:**
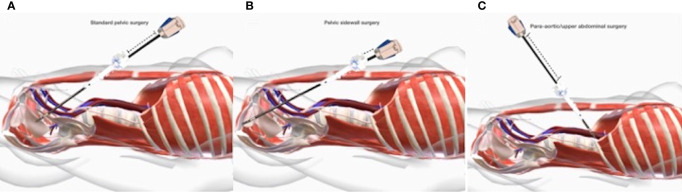
**(A)** Standard pelvic surgery; **(B)** Pelvic sidewall surgery; **(C)** Para aortic/upper abdominal surgery.

In such a situation, the surgeon will perform pelvic surgery limited to the uterus, adnexa, pelvic, and common lymph nodes. For these, the robotic instruments do not have to reach extremely caudal, lateral, ventral, or dorsal surgical targets (**Figure 4A**).

#### 2.2.2 Pelvic Sidewall Surgery

When pelvic sidewall surgery is indicated, the surgeon will mainly drive his surgical maneuvers in retroperitoneal areas in proximity to the pelvis sidewall after an initial intra and retroperitoneal phase. Therefore, given the extremely deep and/or lateral anatomical target, the significantly increased distance from the trocar (the “ground zero”) will have to be entirely covered by the robotic instrument. Typically, these situations match surgery for deep infiltrating endometriosis, neuropelveology, sacrocolpopexy, and deep oncological resections (**Figure 4B**).

#### 2.2.3 Para-Aortic/Upper Abdominal Surgery

In cases of indication for paraaortic/infrarenal surgery or omentectomy as part of surgical staging for gynecological malignancies, after “standard pelvic surgery,” the robotic arms will be driven to surgical targets located cranially to the line of the trocar, thus determining the vertical turnaround of the robotic arms (**Figure 4C**).

### 2.3 Surgical Procedures

The cadaver full bodies were positioned on the operative table in the dorsal lithotomy position, with leg stirrups and shoulder braces to ensure a good Trendelenburg tilt during surgery and replicate the typical space dimensions between the patient and robotic arms.

Once pneumoperitoneum (12 mmHg) was achieved with a Veress needle, a 12 mm robotic trocar was introduced in the transumbilical position.

By considering the aforementioned variables of the robotic arms, positioning, and docking, the surgeons adopted different types of port placement and arm cart allocation. Concerning the port placement, the chosen options were called “straight” and “bridge”; instead, the so-called “compact” and “butterfly” configurations were identified for the arm cart positioning.

For convenience, the left trocar, camera one, the right one, and the right flank trocar will be called 1, 2, 3, and 4, respectively. In the same way, given that the robotic system recognizes the camera arm as number 2, the robotic arms will be clockwise called 1, 2, 3, and 4.

#### 2.3.1 The “Straight” Port Placement

**Figure 5:**
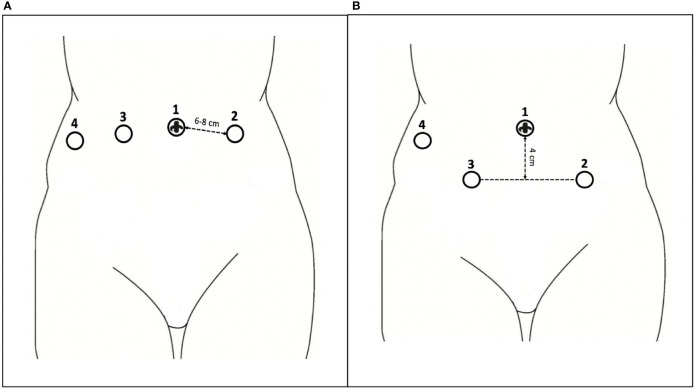
**(A)** The “straight” port placement; **(B)** The “bridge” port placement.

All trocars are introduced along the transumbilical line. Trocar 2 is inserted through the umbilicus. Other robotic trocars (8 mm) are installed as follows: 1 and 3 bilaterally to the umbilical one, at a minimum distance of 6–8 cm, and 4 laterally to the right one, at the level of the flank (**Figure 5A**).

#### 2.3.2 The “Bridge” Port Placement

Unlike the previous proposal, the trocars 1 and 3 are placed symmetrically lateral to the transumbilical one, at a minimum distance of 6–8 cm, but 4 cm lower than the transumbilical line (**Figure 5B**).

#### 2.3.3 The “Compact” Docking Configuration

**Figure 6:**
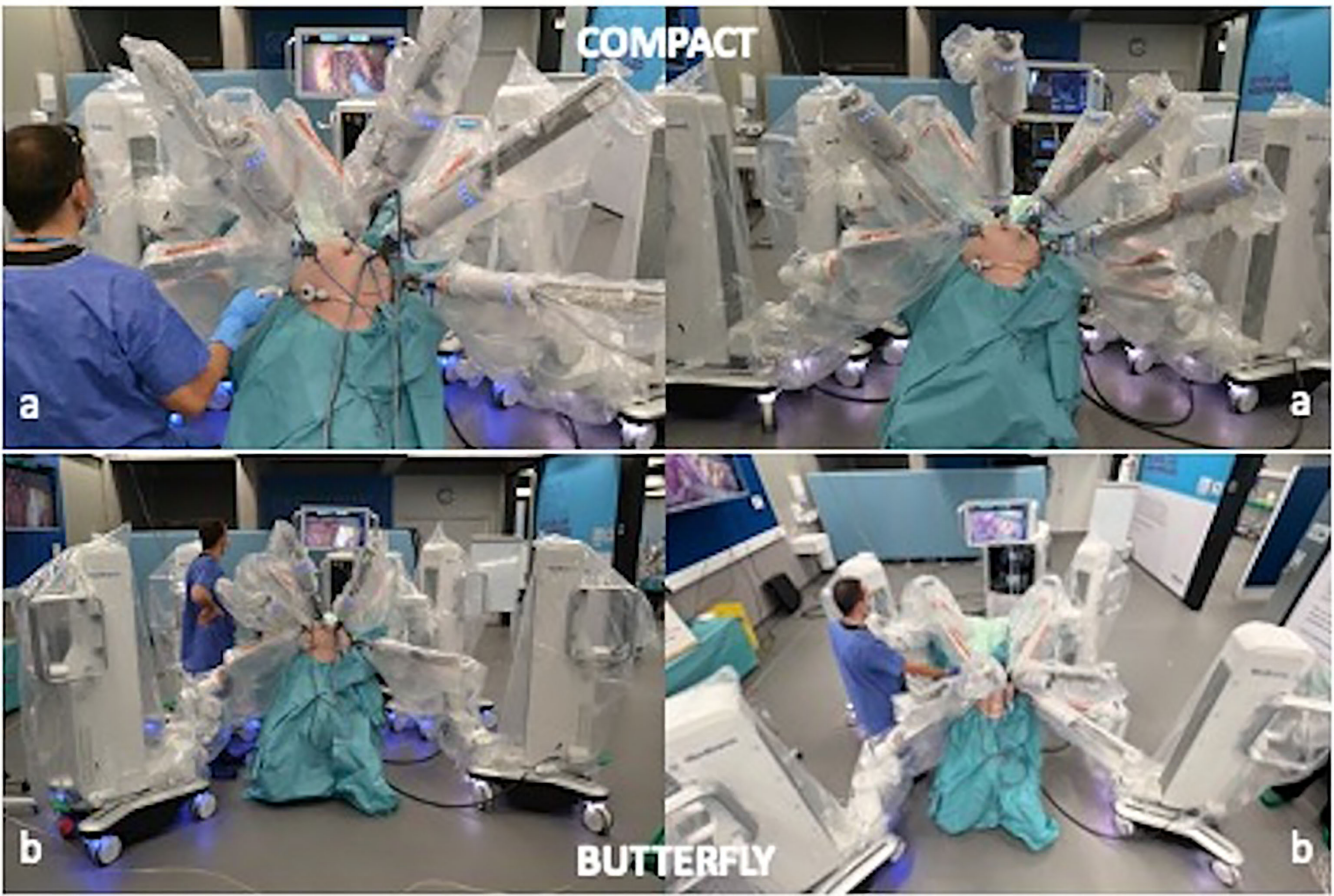
**(A)** The “compact” docking configuration; **(B)** The “butterfly” docking configuration.

The four-arm carts are allocated laterally along the right and left sides of the operative table, two per side. In this configuration, the arm carts are disposed in close contiguity at the level of the legs at an approximate angle of 30° with the operative table (**Figure 6A**). Given the position of the arm carts, the ancillary port for bedside assistant is inserted on Palmer’s point.

#### 2.3.4 The “Butterfly” Docking Configuration

The four-arm carts are disposed, at the four corners of the operative table at an angle of 45° (**Figure 6B**). With this configuration, the bedside assistant can occur between arms 1 and 2 using an ancillary port inserted at the level of the left iliac fossa.

Regardless of both port placement and the docking configuration, a 0° 3D HD camera (Karl Storz) was hooked up on arm 2 and inserted into the transumbilical trocar. Following that, a fenestrated bipolar grasper, a monopolar scissor, and secure Cadiere forceps were hooked on the arms 1, 3, and 4 and inserted on the trocars 1, 3, and 4.

The bed-side assistant used an atraumatic grasper or suction/irrigation device through the ancillary port that, in the presented cases, was a 10 mm Air Seal trocar.

In accordance with the hypotized surgical scenarios, various surgical procedures were performed to assess the described docking setting. Based on the proposed solutions, four different combinations of port placement and docking configuration were used to perform surgery: the compact straight (CS), the compact bridge (CB), the butterfly straight (BS), and the butterfly bridge (BB).

## 3 Results

In total, 4 cadavers were used to perform a wide variety of gynecological procedures. In more depth, total hysterectomy, radical hysterectomy, pelvic exenteration, pelvic and para-aortic lymphadenectomy, and omentectomy were performed to investigate the characteristics and limitations of the different docking settings and, more generally, to investigate the ability of the robotic system to execute the common surgical maneuvers such as traction, dissection, coagulation, and section to open the surgical spaces and move in different anatomical regions.

In the case of compact configuration, after the activation of the laser alignment unit, the robotic arms were hooked to the trocar with the following angles of approach and tilt: arm 1 angle 100° tilt +15, arm 2 angle 140° tilt −30, arm 3 angle 220° tilt −30, and arm 4 angle 260° tilt −15.

When a butterfly configuration was adopted, angles and tilt were set as follows: arm 1 angle 90° tilt +15, arm 2 angle 140° tilt −30, arm 3 angle 220° tilt −30, and arm 4 angle 270° tilt +15.

**Table 1** summarizes the main docking setting variables for both configurations.

**Table 1:** Main docking setting variables for both configurations.

Docking settings	COMPACT		BUTTERFLY	
	Angle (°)	Tilt (°)	Angle (°)	Tilt (°)
**Arm 1**	100	+15	90	+15
**Arm 2 (Camera)**	140	−30	140	−30
**Arm 3**	220	−30	220	−30
**Arm 4**	260	+15	270	+15
**Ancillary port**	Palmer’s Point		Left iliac fossa	

When para-aortic/upper abdominal procedures were performed, either by adopting the compact or the butterfly configuration, we occasionally experienced limited motion of the robotic arms 2 and 3; in these situations, the opposite working direction of the instruments determined the “closure” of each joint of the arm. This limitation was overcome by increasing the negative tilt of arm 2 from −30 to −40 and by reducing one of the arms, 3 from −30 to −20.

In one case of upper abdominal procedure in a low BMI cadaver, an arm cart reallocation was needed to avoid an external collision between arms 3 and 4 due to the proximity of the trocar in a very small abdomen.

The straight port placement revealed itself to be more suitable for standard pelvic and para-aortic/upper abdominal surgery. Due to the length of robotic instruments, comprised of between 52 and 54 cm, the bridge port placement was optimal for the pelvic sidewall procedures.

In the compact docking, the bedside assistant worked seated close to the left shoulder of the cadaver. In this setting, he can assist proficiently in standard pelvic and para-aortic/upper abdominal surgery. Instead, in cases of pelvic sidewall surgery, the increased parallelism between the robotic arm and the abdominal surface determines the risk of hits on the body of the assistant. This problem was overcome when in the butterfly configuration, the assistant took place between arm 1 and arm 2, working with a trocar positioned in the left iliac fossa.

All surgical tasks were successfully completed.

## 4 Discussion

The robotic market is now open, and it has recently been enriched with new surgical robots. All recently launched systems have one main difference with respect to the Da Vinci: compared with the well-known monolithic design, they are based on a modular concept with independent robotic arms.

In this context, it is established that the console and the system tower are constant elements. Each system has specific characteristics and “flexibility” due to the technical design of the robotic arm.

However, flexibility may not always correspond to efficiency in the operative room. Therefore, acquiring specific knowledge is mandatory to avoid logistical, technical, or clinical issues.

From a general standpoint, installing a modular surgical robot into a standard operative room requires some adjustments in the management of the space. Indeed, once installed on the patients, the robotic arms cover an area of about 3 × 4 m around the operative table. Additionally, the driving console and the system tower need to be allocated, respectively, in the corner and at the foot of the operative table. The presence of these new elements in the OR requires a variation in the positioning of the instrument table, the nurse, the anesthesiologist, and, in some conditions, the bedside assistant.

From a clinical perspective, when dealing with a modular surgical robot, the arm cart allocation, the trocar placement, and the docking are fundamental steps in achieving an excellent robotic installation and avoiding external collisions or limitations of movement.

With that said, the variability of the abdomen of each patient and the length of the robotic instruments, once hooked, must be considered leading placeholders in driving the success of the surgical procedure. Therefore, besides the technical aspects of robotic docking, the surgical plan is the fundamental pivotal element; based on the technical characteristics, the docking needs to be personalized in accordance with the surgical procedure.

In our preclinical experience, we performed several tests that identified the best system configurations to draw the proper efficiency from its flexibility in all gynecological surgical scenarios.

The straight port placement seems to be adequate for standard pelvic surgery. In contrast, the bridge trocar position is better for reaching the deeper and lateral anatomical regions of the female pelvis. Even if both settings are easy to move to the upper abdomen without changing any instrument, to vary abdominal conformation, it could be necessary to adjust the tilt angle of the lower arms to maintain a good range of motion and reach the most cranial districts such as the left renal vein.

The compact and the butterfly arm cart allocation are adequate for both the straight and bridge port placements. Again, in our experience, the critical element was the surgical procedure. When deep pelvic surgery was performed, the bedside assistant became more proficient by working with a standard laparoscopic instrument from an ancillary port placed in the left iliac fossa. To do this, the arm carts needed to be moved in an open manner, like for the proposed butterfly configuration. In contrast, the compact disposition left enough space to assist from Palmer’s point port.

Several basic and advanced gynecological surgical procedures were performed and completed successfully without encountering any technical or surgical issues; the results obtained were judged sufficient to proceed with the clinical experience in daily practice.

## 5 Conclusions

As far as we are aware, this is the first paper introducing the novel surgical robot HUGO™ RAS in gynecological surgery (13). Therefore, we wanted to provide a technical description and docking settings from the clinician standpoint. Adopting a new surgical robot means adapting to its surgical environment. In this preclinical experience, the HUGO™ RAS system is flexible and highly performative in various surgical scenarios. However, the proper understanding of its main features and possible limitations represents the key to making a modern, sustainable use of such technology. Even more, a modular robotic system must be considered as an instrument to personalize the surgical treatment for gynecological patients. The surgeon can decide how many robotic arms to use based on the surgical needs. In the case of a full docking, the proposed settings seem to be feasible and efficient for gynecological use. Data from clinical experiences are needed to assess our results further.

## Data Availability Statement

The original contributions presented in the study are included in the article/supplementary material. Further inquiries can be directed to the corresponding author.

## Author Contributions

SGA, VC, GA, and GS contributed to the conception and design of the study. AG, RO, and GM collected technical information and pictures. GS, SGA, AF, and FF wrote the first draft of the manuscript. All authors listed have made a substantial, direct, and intellectual contribution to the work and approved it for publication.

## Conflict of Interest

The authors declare that the research was conducted in the absence of any commercial or financial relationships that could be construed as a potential conflict of interest.

## Publisher’s Note

All claims expressed in this article are solely those of the authors and do not necessarily represent those of their affiliated organizations, or those of the publisher, the editors and the reviewers. Any product that may be evaluated in this article, or claim that may be made by its manufacturer, is not guaranteed or endorsed by the publisher.

## References

[1] Turner LC Shepherd JP Wang L Bunker CH Lowder JL. Hysterectomy Surgery Trends: A More Accurate Depiction of the Last Decade? Am J Obstet Gynecol (2013) 208(4):277.e1–7. doi: 10.1016/j.ajog.2013.01.022 PMC3610857 23333543

[2] Muaddi H Hafid ME Choi WJ Lillie E de Mestral C Nathens A. Clinical Outcomes of Robotic Surgery Compared to Conventional Surgical Approaches (Laparoscopic or Open): A Systematic Overview of Reviews. Ann Surg (2021) 273(3):467–73. doi: 10.1097/SLA.0000000000003915 32398482

[3] Peters BS Armijo PR Krause C Choudhury SA Oleynikov D. Review of Emerging Surgical Robotic Technology. Surg Endosc (2018) 32(4):1636–55. doi: 10.1007/s00464-018-6079-2 29442240

[4] Fanfani F Monterossi G Fagotti A Rossitto C Gueli Alletti S Costantini B. The New Robotic TELELAP ALF-X in Gynecological Surgery: Single-Center Experience. Surg Endosc (2016) 30(1):215–21. doi: 10.1007/s00464-015-4187-9 25840895

[5] Fanfani F Restaino S Gueli Alletti S Fagotti A Monterossi G Rossitto C. TELELAP ALF-X Robotic-Assisted Laparoscopic Hysterectomy: Feasibility and Perioperative Outcomes. J Minim Invasive Gynecol (2015) 22(6):1011–7. doi: 10.1016/j.jmig.2015.05.004 25982854

[6] Gueli Alletti S Rossitto C Cianci S Restaino S Costantini B Fanfani F. TELELAP ALF-X Versus Standard Laparoscopy for the Treatment of Early-Stage Endometrial Cancer: A Single-Institution Retrospective Cohort Study. J Minim Invasive Gynecol (2015) 23(3):378–83. doi: 10.1016/j.jmig.2015.11.006 26602025

[7] Lin CC Huang SC Lin HH Chang SC Chen WS Jiang JK. An Early Experience With the Senhance Surgical Robotic System in Colorectal Surgery: A Single-Institute Study. Int J Med Robot (2021) 17(2):e2206. doi: 10.1002/rcs.2206 33289238

[8] Gueli Alletti S Rossitto C Cianci S Perrone E Pizzacalla S Monterosso G. The Senhance™ Surgical Robotic System ("Senhance") for Total Hysterectomy in Obese Patients: A Pilot Study. J Robot Surg (2018) 12(2):229–34. doi: 10.1007/s11701-017-0718-9 28624984

[9] deBeche-Adams T Eubanks WS de la Fuente SG. Early Experience With the Senhance®-Laparoscopic/Robotic Platform in the US. J Robot Surg (2019) 13(2):357–9. doi: 10.1007/s11701-018-0893-3 30426353

[10] Morton J Hardwick RH Tilney HS Gudgeon AM Jah A Stevens L. Preclinical Evaluation of the Versius Surgical System, a New Robot-Assisted Surgical Device for Use in Minimal Access General and Colorectal Procedures. Surg Endosc (2021) 35(5):2169–77. doi: 10.1007/s00464-020-07622-4 PMC8057987 32405893

[11] Thomas BC Slack M Hussain M Barber N Pradhan A Dinneen E. Preclinical Evaluation of the Versius Surgical System, a New Robot-Assisted Surgical Device for Use in Minimal Access Renal and Prostate Surgery. Eur Urol Focus (2021) 7(2):444–52. doi: 10.1016/j.euf.2020.01.011 32169362

[12] Dixon F Khanna A Vitish-Sharma P Singh NS Nakade K Singh A. Initiation and Feasibility of a Multi-Specialty Minimally Invasive Surgical Programme Using a Novel Robotic System: A Case Series. Int J Surg (2021) 96:106182. doi: 10.1016/j.ijsu.2021.106182 34848372

[13] Ragavan N Bharathkumar S Chirravur P Sankaran S Mottrie A. Evaluation of Hugo RASTM System in Major Urological Surgery: Our Initial Experience. J Endourol (2022). doi: 10.1089/end.2022.0015 35156838

